# Wildlife hunting and the increased risk of leprosy transmission in the tropical Americas: a pathogeographical study

**DOI:** 10.1186/s40249-025-01301-z

**Published:** 2025-05-12

**Authors:** Alisa Aliaga-Samanez, Patricia D. Deps, Julia E. Fa, Raimundo Real, Jean-François Guégan, Marcela A. Oliveira, Aline Pessutti, Simon Knoop, Juliano A. Bogoni, Thais Q. Morcatty, Roberta Marques, Daniel Jiménez-García, Gabriel F. Massocato, Arnaud L. Desbiez, Danilo Kluyber, Hani R. El Bizri

**Affiliations:** 1 Group of Biogeography, Diversity and Conservation, Department of Animal Biology, Faculty of Sciences, University of Malaga, Malaga, Spain; 2 MIVEGEC (UMR University of Montpellier, CNRS, IRD), Montpellier, France; 3 Department of Social Medicine, Postgraduate Program in Infectious Diseases, Federal University of Espírito Santo, Vitória, Brasil; 4 Center for International Forestry Research (CIFOR), Bogor, Indonesia; 5 Department of Natural Science, Manchester Metropolitan University, Manchester, UK; 6 Natural Sciences and Environment Hub, University of Gibraltar, Gibraltar, Gibraltar; 7 Institute of Biotechnology and Blue Development (IBYDA), University of Malaga, Malaga, Spain; 8 Research Network On Diversity, Conservation and Use of Wildlife in Amazonia (RedeFauna), Manaus, Brazil; 9 Postraduate Program in Conservation and Use of Natural Resources, Federal University of Rondônia, Porto Velho, Rondônia Brazil; 10 Department of Biology, University of Oxford, Oxford, UK; 11 Sainsbury Wellcome Centre, University College London, London, UK; 12 Living Gaia E.V., Berlin, Germany; 13 Postgraduate Program in Environmental Sciences, Limnology, Biodiversity, and Ethnobiology Research Center of Pantanal, Mammalogy Laboratory, State University of Mato Grosso, Cáceres, MT Brazil; 14 Department of Geography, University College London, London, UK; 15 Oxford Wildlife Trade Research Group, Faculty of Humanities and Social Sciences, Oxford Brookes University, Oxford, UK; 16 Biodiversity Laboratory, Agroecology and Environment Center, Institute of Sciences, Benemérita Autonomous University of Puebla, Puebla, Mexico; 17 Wildlife Conservation Institute (ICAS), Campo Grande, Mato Grosso do Sul Brazil; 18 Houston Zoo, Houston, TX USA; 19 Institute for Ecological Research (IPÊ), Nazaré Paulista, Brazil; 20 RZSS, The Royal Zoological Society of Scotland, Edinburgh, UK; 21 Faculty of Medicine, University of São Paulo, São Paulo, Brazil; 22 Research Group on Terrestrial Vertebrate Ecology, Mamirauá Sustainable Development Institute, Tefé, Brazil

**Keywords:** Disease ecology, Human-animal interaction, Armadillo, *Mycobacterium leprae*, Zoonotic diseases, Pathogeography

## Abstract

**Background:**

Leprosy remains a persistent public health challenge, where human-to-human transmission of *Mycobacterium leprae* via respiratory droplets is well established. In the tropical Americas, growing evidence implicates armadillos as important zoonotic reservoirs, particularly through direct contact during hunting and handling. However, such transmission has so far been considered rare and highly localised. This study provides a comprehensive spatial analysis of the role of armadillo hunting in human leprosy transmission, quantifying its contribution to disease prevalence and identifying geographic hotspots where interventions could be most effective.

**Methods:**

Using Brazil’s 326,001 reported leprosy cases from 2013 to 2022, we applied a pathogeographical approach to explore transmission dynamics. We compiled data on 554 hunted armadillos across 175 municipalities and *M. leprae* prevalence in 376 armadillo individuals from 97 municipalities (mean prevalence = 38.5%). These were used to build spatial models assessing hunting-related infection risk and integrated as a variable into a generalised linear model alongside socioeconomic, climatic, and environmental predictors to evaluate their effects on human leprosy prevalence.

**Results:**

Key predictors of armadillo hunting included higher population density (*P* < 0.001) and firearm availability (*P* < 0.01). Infection in armadillos was negatively correlated with native habitat coverage (coefficient: − 2.28;* P* < 0.001), suggesting that environmental degradation can amplify infection risk. The armadillo-hunting infection risk variable—generated by combining armadillo hunting and infection favourability models—emerged as the second strongest predictor of human leprosy prevalence (coefficient: 1.69; *P* < 0.001), accounting for ~ 25% of cases nationally and around 40% in deforestation hotspots. Additional positive predictors included greater precipitation seasonality (coefficient: 0.82; *P* < 0.001) and malnutrition (coefficient: 0.01; *P* < 0.001), while higher population density (coefficient: − 0.64; *P* < 0.001), natural habitat coverage (coefficient: − 0.50; *P* < 0.001) and socioeconomic status (coefficient: − 0.47; *P* = 0.013) were linked to reduced disease prevalence.

**Conclusions:**

Armadillo hunting seems to play a more significant role in human leprosy transmission than previously recognised. To address this overlooked pathway, targeted interventions should focus on reducing unsafe and illegal hunting, improving communication around zoonotic risks, strengthening disease surveillance in high-risk areas, and conducting genetic studies to confirm wildlife-to-human transmission. Our findings highlight the importance of incorporating wildlife-associated transmission pathways into strategies to reduce disease prevalence and mitigate future outbreaks in tropical regions facing rapid environmental change and persistent poverty.

**Graphical Abstract:**

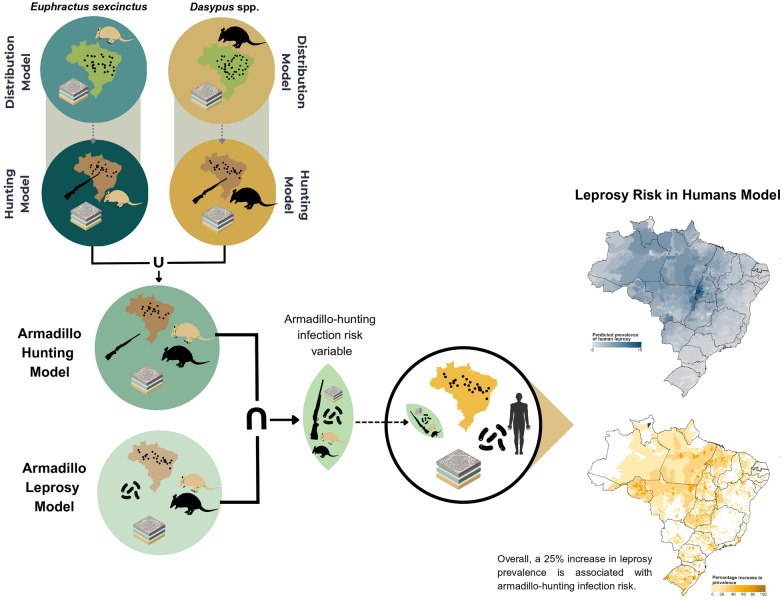

**Supplementary Information:**

The online version contains supplementary material available at 10.1186/s40249-025-01301-z.

## Background

The COVID-19 pandemic has highlighted the urgent need for rapid and accurate monitoring of disease emergence risks regionally and globally. Effective monitoring and real-time data acquisition are crucial for informed decision-making, reducing human suffering and limiting economic disruptions [1, 2]. Understanding and mapping the spatio-temporal dynamics of diseases has become essential for improving prediction and response strategies as these increasingly spread across borders [3, 4]. Recent research emphasises the importance of advancing methods and developing innovative conceptual frameworks for tracking the spread of zoonotic diseases. Disease ecology addresses how species interactions, including host–pathogen relationships and environmental conditions, affect the processes of disease emergence and spread [5]. Pathogeographical models, which incorporate key predictive variables—including host reservoirs, vectors, environmental, social, and spatial factors, and human exposure and vulnerability—offer a comprehensive tool for capturing the complexity of disease life-cycles and improving disease management efforts [6–9].

The resurgence of mycobacteria, particularly *Mycobacterium leprae* and *M. tuberculosis*, which cause leprosy and human tuberculosis, respectively, poses a significant public health threat both regionally and globally. Recent waves of mass human migration coupled with global environmental changes such as climate warming have increased the risk of these pathogens re-emerging in regions where they were previously controlled or eliminated [10]. Leprosy, also known as Hansen’s disease, is a transmissible, debilitating and socially stigmatising illness. In 2022 alone, 174,087 new cases were reported across 128 countries, with India, Brazil, and Indonesia accounting for 78.1% of the global total. Despite its prevalence, leprosy remains classified by the World Health Organization (WHO) as a neglected tropical disease (NTD), primarily affecting populations in tropical and subtropical regions. Its transmission is closely linked to poverty [11–13], nutritional deficiencies, prolonged exposure to infected individuals, geographical disparities and limited access to health care [14].

Leprosy transmission primarily occurs from untreated persons with the multibacillary form of the disease, which is characterized by higher bacterial loads, making these patients the primary source of *M. leprae* [15]. The bacillus is mainly spread via droplets from the upper respiratory tract, although transmission through skin lesions is also possible. In contrast, the paucibacillary form, associated with significantly lower bacterial loads, presents a much lower risk of transmission. While human-to-human transmission remains the dominant pathway, environmental sources of *M. leprae* have been explored since the early twentieth century [16, 17]. The WHO’s Global Leprosy Strategy 2021–2030 provides comprehensive guidance to interrupt leprosy transmission and ultimately eliminate the disease [15]. This guidance acknowledges that zoonotic transmission of *M. leprae* from contact with armadillos (mammals of the order Cingulata) has been demonstrated, though the risk has so far appeared to be low and highly localised [17]. Currently, there is no evidence of transmission from other known animal reservoirs. However, recent studies suggest that exposure to armadillos is associated with a significantly elevated infection risk, indicating that they may serve as plausible reservoirs and play a more important role in transmission than previously recognised [18–21].

Multiple case–control studies conducted in the USA, Brazil, and Colombia consistently show a strong association between direct contact with wild armadillos and an elevated risk of contracting leprosy**,** especially among hunters and consumers of these animals [18, 19, 21–25]. Natural infection with *M. leprae* in armadillos was first reported in Brazil by Deps et al. (2002) [26] in 2002, with approximately 1 in 10 armadillos found to be infected [27]. Although a second *Mycobacterium* species close to *M. leprae*, i.e., *M. lepromatosis,* was identified as a causal agent of leprosy-like symptoms in 2008 [28], this pathogen has not yet been detected in animal reservoirs anywhere in the world [29].

In Brazil, where armadillo hunting is widespread [30, 31] and leprosy remains a persistent public health concern across the country, approximately 28,000 new leprosy cases are detected annually. Individuals involved in hunting, preparing, and consuming armadillos, face nearly double the risk of developing leprosy compared to those with no direct contact [18, 27]. While leprosy transmission through armadillo contact has been recognised in the USA, a non-endemic country where leprosy is officially acknowledged as a zoonotic disease after a combination of epidemiological studies and genetic analyses [32, 33], efforts in Brazil have focused mainly on human-to-human transmission, neglecting the potential contribution of zoonotic modes and pathways [34]. This limited attention to zoonotic sources complicates the development of effective, targeted interventions to address the role of wildlife-associated transmission in the tropical Americas.

In response, this study employs a disease ecology modelling approach to investigate the dynamics of leprosy transmission linked to armadillo hunting using Brazil as a representative case for the tropical Americas, given its large geographical coverage and high disease incidence. By using pathogeographical models, we aim to provide the most comprehensive assessment to date of how armadillo hunting and associated practices might contribute to leprosy transmission in humans. These insights will help inform the development of more tailored public health interventions that account for both zoonotic and human-to-human transmission pathways, and their respective roles in endemic regions across the tropical Americas and beyond. In addition, our findings will support the assessment of whether revisions to current health guidance on zoonotic transmission of leprosy are warranted.

## Methods

### Study area

Brazil’s tropical area, largely covered by the Amazon basin, represents roughly 40% of the total surface of tropical Americas, where armadillos occur and are widely hunted [30, 31]. All datasets covering human leprosy cases (DATASUS), *M. leprae* infection in armadillos (from published studies), and armadillo hunting observations used in this study were available at the municipality scale only. This aggregation reflects privacy protocols for health data, common reporting practices in the literature, and the frequent absence of precise geographic coordinates for hunting records. Accordingly, we standardised our analyses at the municipality level in Brazil, including the explanatory environmental, socioeconomic and spatial variables. For certain environmental variables, mean values per municipality were computed using ArcMAP 10.7 software (Esri, Redlands, CA, USA). A comprehensive list of these variables is available in Table S1, Additional file 1.

### Methodological framework

This study primarily developed spatially explicit models that quantify the risk of human leprosy across Brazil and evaluated the significance of armadillo hunting as a contributing factor to the incidence of new human leprosy cases nationwide. We also estimated the proportion of leprosy cases attributable to armadillo hunting, and identified the regions in Brazil where this relationship is most pronounced, and people are more at risk to contracting this disease through wildlife hunting.

**Fig. 1:**
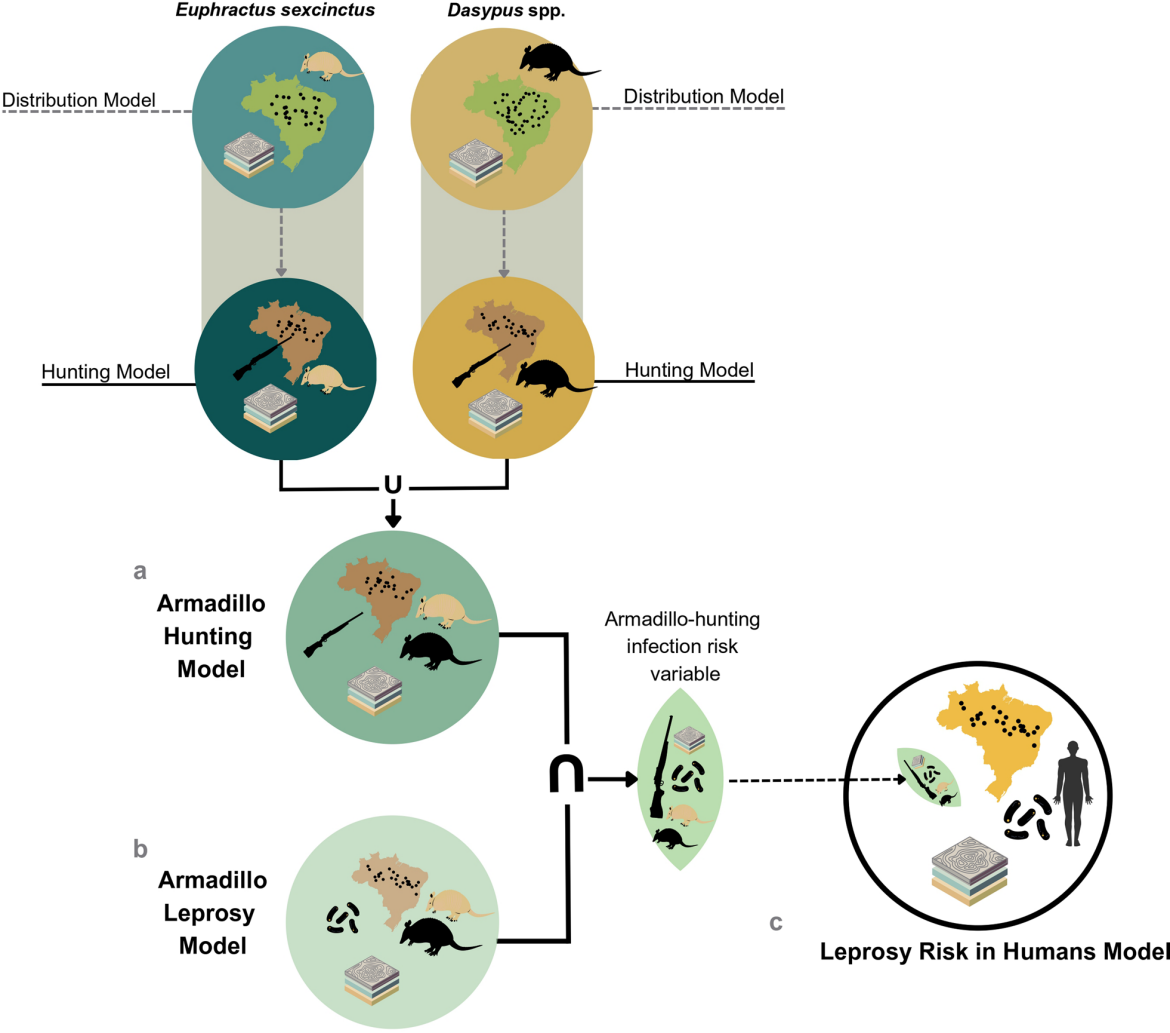
Methodological framework for leprosy transmission risk modelling from armadillo taxa to humans used in this study. a. Armadillo Hunting Model, b. Armadillo Leprosy Model, and c. Leprosy Risk in Humans Model

Our methodology consisted of three main steps, as illustrated in Fig. 1:

1. *Armadillo Hunting Model*: We first identified areas in Brazil where armadillos were most hunted.
2. *Armadillo Leprosy Model:* Then we identified areas where there were records of prevalence of *M. leprae* in armadillos to assess the risk of infection due to armadillo hunting; and
3. *Leprosy Risk in Humans Model*: We integrated the infection risk due to armadillo hunting as a variable in a broader model assessing human leprosy risk.

All models included their own set of environmental, socioeconomic and spatial predictors (see Table S1, Additional file 1).

For all models involving armadillos, we used records of two taxa (see Fig. S1, Additional file 1): six-banded armadillo (*Euphractus sexcinctus*) and long-nosed armadillos (*Dasypus* spp.). These taxa were selected based on their documented associations with *M. leprae* in the literature—they are the only armadillo taxa for which the bacterium was detected apart from a single recent detection in *Cabassous tatouay *[27, 29]. We treated *Dasypus* at the genus level because recent studies indicate that species classification within the genus (e.g., *Dasypus hybridus*, *Dasypus beniensis*) cannot be reliably determined through morphology alone, requiring molecular investigations for accurate delimitation [35–37]. This suggests that, although the nine-banded armadillo (*Dasypus novemcinctus*) is the most frequently reported species in distribution and hunting records, as well as studies on *M. leprae* infection, these records likely encompass multiple *Dasypus* species. Given their similar external morphology, physiology, and genetic relatedness, particularly as *Dasypus* is the only extant genus within the Dasypodidae family, it is reasonable to expect that susceptibility to infections is broadly consistent across these species. This expectation is supported by studies demonstrating that pathogen susceptibility in mammals is often phylogenetically conserved [38]; and research on *Mycobacterium* phylogeny showing that virulent species such as *M. leprae* tend to infect phylogenetically clustered mammalian hosts [38].

### Armadillo hunting model

We compiled occurrence records of *Euphractus sexcinctus* and *Dasypus* spp. to build species distribution models [39–44]. We defined a set of spatial, socioeconomic, and environmental variables to explain the distribution of these taxa (see Additional file 1) which was included into two independent distribution models, one for each armadillo taxon. To ensure the robustness of our spatial models, we implemented controls during the distribution modelling process. First, we evaluated the relationship between each explanatory variable and the presence (1) or absence (0) of occurrence records for each armadillo taxon in each municipality using binary logistic generalised linear model (GLM), a supervised machine learning algorithm commonly used in species distribution modelling. Only significant explanatory variables, determined by Rao's score (RS) test were retained, which estimated the significance of their association with the distribution of armadillos. To account for the increased risk of Type-I errors associated with a more extensive set of independent explanatory variables, we applied the false discovery rate (FDR) method [45], selecting only those variables considered significant under an FDR threshold of *q* < 0.05 for subsequent multivariate ensemble models.

These multivariate explanatory models were also developed using binary logistic regression, with variable selection conducted through a forward–backward stepwise approach. In the forward step, variables were added to the model based on their significance and contribution to explaining the variability in taxa occurrence, starting with the most significant predictor. Conversely, during the backward step procedure, the least significant variables were iteratively removed one at a time.

To mitigate excessive multicollinearity in the multivariate models, we ensured that variables with Spearman’s correlation coefficients > 0.8 were not included in the same model [9]. If this occurred, the least significant variable was removed, and the stepwise procedure was re-run.

In this way, the probability of occurrence of each armadillo taxon in each Brazilian municipality was obtained. We then ran, for each taxon, the Favourability Function [46, 47]:

1 $$F=\frac{P}{1-P}/\left(\frac{{n}_{1}}{{n}_{0}}+\frac{P}{1-P}\right)$$

where *F* is the favourability of occurrence for each taxon in each municipality, n_1_ and n_0_ are the number of municipalities with and without the occurrence for each taxon, respectively, and *P* is the probability of occurrence of each armadillo taxon in each municipality. Outputs and favourability maps for *E. sexcinctus* and *Dasypus* spp. distribution models can be found in the Additional file 1.

The scores obtained in these favourability models served as predictor variables in analysing the spatial distribution of armadillo hunting occurrences for each taxon. To build the hunting models, we used hunting records from a comprehensive study in Brazil that used social media posts to record hunting activities conducted between 2018 and 2020, documenting 554 armadillo individuals hunted across 175 municipalities [31]. These records primarily captured instances of illegal sport hunting where armadillo meat is often consumed post-hunt. The dataset provided information on hunting occurrences, species targeted, and associated practices (e.g., hunting, handling, and consumption). Further details on the data collection and methodologies for hunting data can be found in El Bizri et al. (2024) [31].

The probability of hunting each armadillo taxon at each municipality was obtained using binary logistic regression. We treated the presence (1) or absence (0) of hunting records for each taxon in each municipality as the dependent variable. A new set of environmental, socioeconomic and spatial descriptors served as independent predictor variables (see Additional file 1). The assessment of the explanatory capacity of each variable and the control of the FDR and multicollinearity was performed as explained above for the distribution models. Since hunting can only occur in areas where armadillos are present, the results from the distribution models for each armadillo taxon were included with forced entry in the ensemble multivariate models, thereby ensuring our model accurately reflected the conditional nature of hunting probabilities. All the other independent variables were selected  using the backward-forward stepwise approach.

To obtain the degree with which each municipality is favourable for hunting armadillos, we used the Favourability Function of Eq. (1) where *F* is the favourability of each taxon being hunted at each municipality, n_1_ and n_0_ are the number of municipalities with and without hunting reports for each armadillo taxon, respectively, and *P* is the probability of hunting for each armadillo taxon in each municipality. The hunting models for each taxon were combined through a fuzzy union to obtain a single Armadillo Hunting Model (Fig. 1a).

The distribution models for each taxon, the hunting models for each taxon, and the single Armadillo Hunting Model were evaluated based on their favourability values’ classification and discrimination capabilities. Six classification assessment indices were used [48]: (1) sensitivity (proportion of the number of municipalities with positive cases correctly classified as favourable), (2) specificity (proportion of the number of municipalities with negative cases correctly classified as unfavourable), (3) Correct Classification Rate (proportion of municipalities, either with positive or negative cases, correctly classified), (4) True Skill Statistics (sensitivity + specificity—1), which measures the overall classification performance of the model, (5) underprediction rate (proportion of unfavourable municipalities with positive cases), and (6) overprediction rate (proportion of favourable municipalities with negative cases). Discrimination ability was assessed according to the area under the receiver operating characteristic (ROC) curve (AUC) [49].

### Armadillo leprosy model

We built a database of instances of detection of *M. leprae* in armadillos available in the literature (see Deps et al. 2020 [27] and Monsalve-Lara et al. 2024 [29]). We considered data on the presence of *M. leprae* in armadillos obtained in 97 municipalities, comprising 10 studies conducted between 2002 and 2024, involving 376 armadillos, with a mean prevalence of 38.5% per municipality (see Additional file 2).

We first calculated the prevalence in terms of proportion of individuals positive for *M. leprae* in each surveyed municipality. We then ran a model using the prevalence as the dependent variable and a set of environmental descriptors as independent predictor variables. We used a generalised linear mixed model (GLMM) under a Beta-Inflated family of distribution (link = Logit), considering that the response variable is bounded between 0 and 1. While the standard Beta distribution excludes exact 0 and 1 values, the Beta-Inflated distribution allows the inclusion of these boundary points in the response variable. This feature makes the Beta-Inflated distribution particularly useful for modelling data that contain a mix of continuous proportions and exact boundary values often encountered in epidemiological studies. This model included the study source (different studies) as a random effect to control for inter-study variability and enhance the generalizability of our results across diverse study conditions, particularly differences in terms of time-period of data collection and methodologies used to determine the presence of *M. leprae* in armadillos (see Deps et al. 2020 [27]). Additionally, we applied weights to adjust for the varying sample sizes per municipality regarding the number of armadillos examined and compensate for potential over- or under-sampling across different studies, ensuring a more balanced representation from each municipality.

We checked for multicollinearity using the Variance Inflation Factor (VIF), considering VIF values lower than 5 for each variable as representing no relevant collinearity [50]. Variables with scores above this threshold were removed from the analyses. Variable selection was implemented through a forward–backward stepwise approach. Each addition and removal of variables were evaluated using Akaike Information Criterion (AIC) to evaluate model improvement. This iterative process continued until only variables contributing to the best model fit (i.e., the model with the lowest AIC) were retained. *P*-values for each retained predictive variable were calculated within the GLMM to determine the statistical significance of their association with the prevalence of *M. leprae* in armadillos in the municipalities (*P*-value < 0.05 was considered significant). The model was run in R Studio Version 2023.09.1 + 494 (RStudio, PBC, Boston, MA) using the *gamlss* (glmm) and *car* (VIF analysis) packages.

We then extrapolated our final model to municipalities where we lacked data on prevalence. We applied the Favourability Function of Eq. (1), where *F* is the degree to which each municipality is favourable for armadillos being infected by *M. leprae*, n_1_ and n_0_ are the number of analysed individuals infected and not infected by *M. leprae*, respectively, and *P* is the predicted prevalence in terms of the proportion of armadillos positive for *M. leprae* in each municipality. These data were utilised to generate a spatial map illustrating favourability scores across all municipalities.

### Armadillo-hunting infection risk variable

We used the Armadillo Hunting Model (Fig. 1a) and the Armadillo Leprosy Model (Fig. 1b) to create a variable reflecting the likelihood of a hunter killing, butchering, and consuming an armadillo infected with *M. leprae* (hereafter *armadillo-hunting infection risk*). We considered that hunters were consuming armadillos themselves because most of the hunting records portrayed the animal being cooked. To this aim, the hunting model and the leprosy model were combined through a fuzzy intersection to obtain the armadillo-hunting infection risk in each municipality (Fig. 1). This risk was used as an explanatory variable in the Leprosy Risk in Humans Model (see below; Fig. 1c).

### Leprosy risk in humans model

Data on human leprosy cases from 2013 to 2022 were sourced from the Brazilian Ministry of Health’s DATASUS program [51], totalling 326,001 registered cases across Brazil. We conducted a GLM under a Beta-Inflated family of distribution (link = Logit), considering that the response variable is bounded between 0 and 1, to evaluate factors influencing the prevalence of leprosy in humans. Prevalence rates were calculated as the *per capita* number of leprosy cases per year (number of new leprosy cases/number of inhabitants/10) for each municipality.

We checked for multicollinearity using VIF, and variable selection was implemented through a forward–backward stepwise approach, assessed using AIC; the lower the AIC score, the better fitted the model. *P*-values for each retained predictive variable were calculated within the GLM to determine the statistical significance of their association with the prevalence of leprosy in humans in (*P*-value < 0.05 was considered significant). We also calculated the model’s generalised coefficient of determination, i.e., R^2^, which refers to the variability in the response variable that is accounted for by the predictors included in the model.

We subsequently produced a final predictive map illustrating the expected leprosy prevalence per 10,000 inhabitants across municipalities based on the coefficients of the variables retained in the model. The model was run in R Studio using the *gamlss* (glm) and *car* (VIF analysis) packages, while the maps were produced using the *sf* and *ggspatial* packages.

### Contribution and hotspots of armadillo-hunting infection risk

We conducted a partition analysis of variance [52] to isolate the specific contribution of the armadillo-hunting infection risk factor to the occurrence of human leprosy cases. This analysis estimated the proportion of variation in the likelihood of human leprosy occurrence attributable solely to the risk posed by armadillo hunting, distinguishing it from the effects of other factors.

To do so, we first removed the armadillo hunting infection risk variable from the 'Risk of Leprosy in Humans Model'. We ran this new model, which contained only the remaining socioeconomic, environmental, and spatial variables. The new model's outcomes (estimated prevalence) were linearly regressed against the leprosy prevalence calculated from the original model that included hunting for each municipality. The residuals from this regression were used as a measure of the specific contribution of armadillo hunting to infection in each municipality. Residuals were quantified as a percentage by dividing them by the final model values and finally multiplying them by 100. We focused on the positive percentages to identify areas with the highest risk due to armadillo hunting and mapped these areas to highlight regions where the risk posed by armadillo hunting is particularly pronounced, regardless of other influencing factors.

## Results

### Armadillo hunting model

**Fig. 2:**
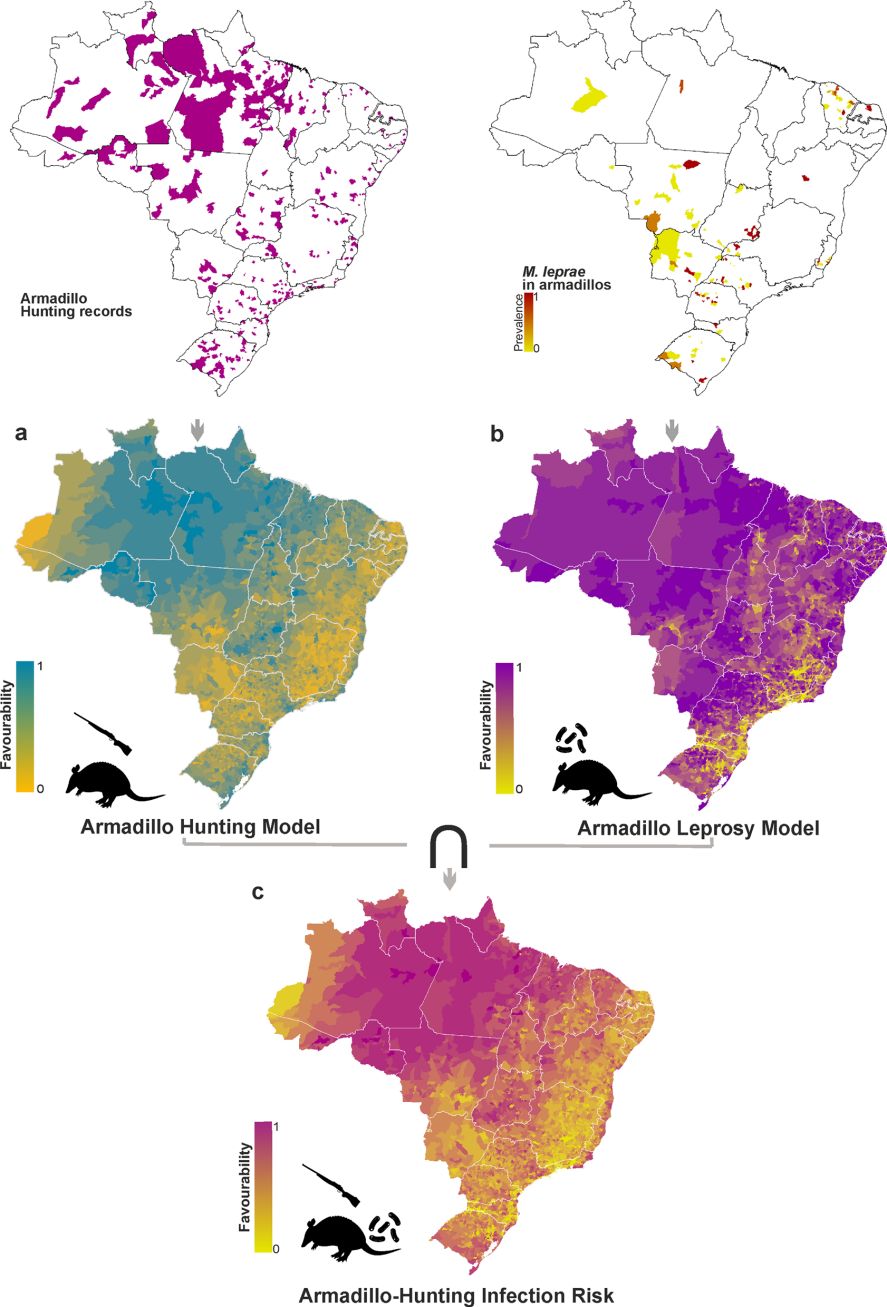
Armadillo-hunting infection risk variable. **a** Armadillo Hunting Model **b** Armadillo Leprosy Model. The risk of contact with armadillo infected through hunting (armadillo-hunting infection risk) **(c)** is estimated as the intersection ( ∩) between conditions favourable for armadillo hunting and conditions favourable for armadillo infection with *M. leprae*. Spatial resolution is at the level of municipalities. Hunting records of *Dasypus* spp. and *Euphractus sexcinctus* and leprosy prevalence data in armadillos are also mapped

The outputs of the distribution models of *E. sexcinctus* and *Dasypus* spp., which were the first step to build the Armadillo Hunting Model, can be seen in Fig. S2, Additional file 1. The Armadillo Hunting Model reveals that the most favourable areas are in the Amazon basin, especially the northern, north-eastern, and central-western regions, as well as specific coastal areas of the Atlantic Forest and parts of the southern Pampa biomes (Fig. 2a).

Higher human population density was a key factor influencing the hunting of both armadillo taxa (*P* < 0.001), likely linked to a greater number of people practicing hunting. For *Dasypus* spp., the model further indicated the significance of environmental factors such as lower road density (coefficient: − 1.41; *P* = 0.011) and gentler terrain slopes (coefficient: − 0.26; *P* < 0.01), which increased the favourability of armadillo hunting. Moreover, higher proportion of guns per civilian within municipalities proved to be a relevant driver of higher armadillo hunting favourability for both species (Table S2, Additional file 1). Classification and discrimination performance of the model can be seen in Table S3, Additional file 1.

### Armadillo leprosy model

The Armadillo Leprosy Model revealed a strong negative association with road density (coefficient: − 0.83; *P* < 0.001), suggesting that areas with higher road density correlated with a lower prevalence of *M. leprae* in armadillos. Sparse vegetation was associated with a higher prevalence of armadillos infected with *M. leprae*. Areas with a greater mix of natural vegetation and cropland, such as those with a mixture of grassland and woodland/shrubland, were associated with a lower prevalence (coefficient: − 1.43; *P* < 0.001), while areas with higher temperature annual range (coefficient: 0.67; *P* < 0.001) and maximum temperature of the warmest month (coefficient: 34.24; *P* < 0.001) were associated with higher prevalence. In addition, areas with higher proportion of remaining native habitat coverage were associated with a lower prevalence (coefficient: − 2.28; *P* < 0.001). This negative association implies that more intact and undisturbed natural environments are related to reduced prevalence of infection in armadillos (Table S4, Additional file 1).

### Armadillo-hunting infection risk

Regions across the country exhibited high and intermediate levels of favourability for armadillos to contract *M. leprae*, except some areas of the Atlantic Forest and in the Caatinga ecoregion of the north-east (Fig. 2b). In states such as Mato Grosso, Pará, Maranhão, Ceará and Rio Grande do Norte, which showed higher prevalence of leprosy in armadillos, the favourability for the risk of contact with an infected armadillo due to hunting is high (*F* > 0.5) (Fig. 2c). The exceptions include municipalities from Rio de Janeiro, Santa Catarina, and Minas Gerais states, where the favourability is generally low (*F* < 0.2) to low-intermediate (0.2 > *F* > 0.5).

### Leprosy risk in humans model

A combination of environmental and socioeconomic factors significantly influenced the prevalence of leprosy in humans, with the model explaining 30% of the variability in the prevalence (generalised R^2^ = 0.30). Human population density was negatively associated with the prevalence of leprosy, presenting the highest Wald’s test value (Table 1). The armadillo-hunting leprosy risk variable presented the second-highest Wald’s value, showing a strong positive association with leprosy prevalence. Additionally, factors such as precipitation seasonality and the extent of closed broadleaved deciduous forests were positively associated with increased leprosy prevalence, suggesting that particular ecosystems and climatic conditions may contribute to the persistence of the disease in some areas (Table 1).

**Table 1:** Leprosy Risk in Humans Model outputs showing the influence of environmental and socioeconomic variables on the incidence of leprosy in humans

Variable	Coefficient	Standard error	*t*-value	Wald’s value	*P*-value
Intercept	− 7.96	0.37	− 21.41	458.56	< 0.001
Human population density (log10)	− 0.64	0.02	− 29.98	898.65	< 0.001
Armadillo-hunting infection risk	1.69	0.06	28.34	803.32	< 0.001
Precipitation seasonality (coefficient of variation)	0.82	0.06	14.48	209.61	< 0.001
Closed broadleaved deciduous forest (> 40%)	2.05	0.17	11.95	142.68	< 0.001
Natural habitat coverage	− 0.50	0.05	− 9.40	88.36	< 0.001
Density of rivers	1.39	0.15	9.36	87.62	< 0.001
Closed to open grassland	− 29.58	3.89	− 7.61	57.84	< 0.001
Temperature annual range	− 1.11	0.15	− 7.59	57.61	< 0.001
Malnutrition	0.01	0	4.90	23.97	< 0.001
Closed to open vegetation (grassland/shrubland) on regularly flooded or waterlogged soil	1.74	0.37	4.77	22.74	< 0.001
Closed broadleaved semi-deciduous or evergreen forest regularly flooded—saline water	− 0.88	0.34	− 2.58	6.67	0.010
Human Development Index	− 0.47	0.19	− 2.48	6.15	0.013
Closed to open shrubland	− 0.15	0.09	− 1.70	2.88	0.090
Closed to open (> 15%) broadleaved evergreen or semi-deciduous forest	− 0.11	0.07	− 1.48	2.18	0.140

Variables are ranked by their Wald’s statistics in descending order, indicating their relative importance in predicting leprosy prevalence in humans

**Fig. 3:**
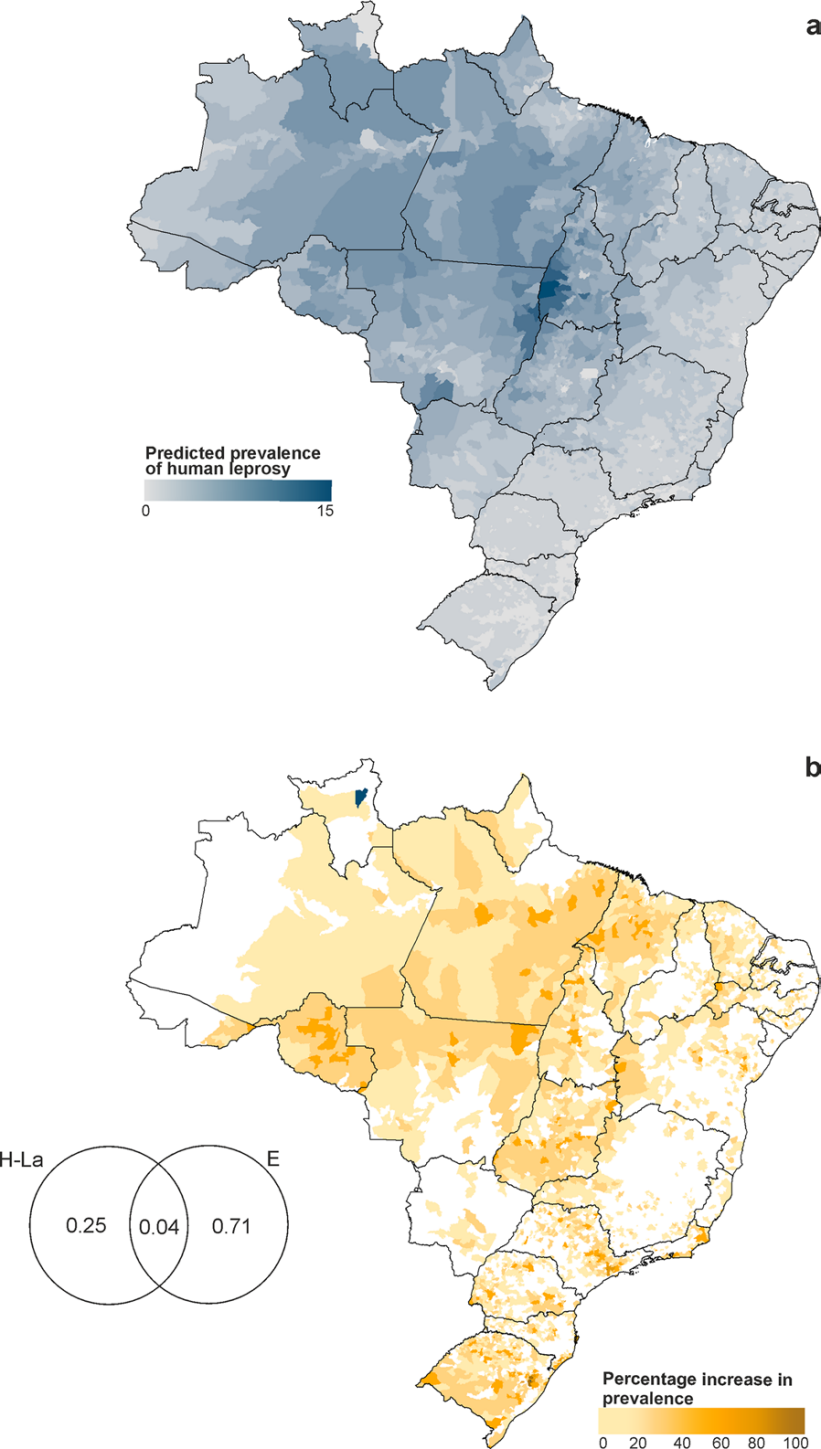
Human leprosy risk and potential areas of influence of the risk of contact with infected armadillo through hunting on human leprosy prevalence.** a** Predicted prevalence of human leprosy where the number of people per 100,000 likely to contract leprosy is represented on the map; **b** areas with increased leprosy prevalence due to armadillo-hunting infection risk, plotted as a percentage. Venn diagrams: numbers are percentage contributions to the expected values in the Leprosy Risk in Humans Model (H-La: risk of contact with infected armadillos through hunting; E: environmental factor). In **b**, the two blue-coloured municipalities are those where the percentage is greater than 100

Conversely, both natural habitat coverage and temperature annual range showed negative associations with leprosy prevalence. Socioeconomic factors also played key roles; areas with higher malnutrition rates showed a higher prevalence of leprosy, while regions with higher Human Development Index (indicative of better socioeconomic conditions) were linked to lower leprosy prevalence. In municipalities in Mato Grosso, Tocantins and Pará states, around 15 out of every 10,000 inhabitants were likely to contract leprosy (Fig. 3a). The Atlantic Forest and Cerrado biomes were the least likely regions for humans to be infected with *M. leprae* (Fig. 3a).

### Contribution and hotspots of armadillo-hunting infection risk

The risk of infection by armadillo hunting accounted for a maximum of 29% of the geographic variation in the prevalence of leprosy in humans (Fig. 3), with 25% of this variation attributable solely to this factor (H-La in Fig. 3). Figure 3 shows the areas where the prevalence increased explicitly due to the influence of armadillo hunting. Between 2013 and 2022, particularly in the Amazon region called “arc of deforestation” located in states such as Rondônia, Mato Grosso, Pará and Maranhão, contact with infected armadillos through hunting contributed to around 40% to human leprosy cases, likely due to a combination of high hunting and deforestation rates, and high number of armadillos infected in those areas (Fig. 3). Areas further south in Goiás and Rio Grande do Sul states also presented elevated contributions of armadillo-hunting infection risk to human leprosy prevalence.

## Discussion

This study significantly advances our understanding of armadillo hunting dynamics and its implications for human leprosy transmission in the tropical Americas. Using Brazil as a model and leveraging the most comprehensive and consistent database of primary hunting records to our knowledge, we mapped hunting likelihood across the country and estimated its contribution to leprosy prevalence in the human population. Our findings indicate that armadillo hunting is a key risk factor and seems to play a more substantial role in human leprosy transmission across the tropical Americas than previously recognised. Additionally, we identified specific regions as hotspots for leprosy infections linked to armadillo hunting, reflecting broader patterns that may apply to other tropical regions in the Americas.

Our reliance on municipality-level, cross-sectional data enabled us to examine broad-scale patterns of leprosy transmission across Brazil in a systematic, comparable manner. We integrated publicly available datasets that consistently report human cases, *M. leprae* infection in armadillos, and hunting records at the municipality level to identify macroecological drivers and potential hotspots that might remain undetected in finer-scale or single-site studies. We nevertheless acknowledge that such an approach cannot resolve local heterogeneities or temporal fluctuations in *M. leprae* transmission dynamics. Future research would benefit greatly from high-resolution spatial data (e.g., precise hunting and armadillo infection locations) and longitudinal observations (e.g., multi-year surveillance of armadillo infections) to strengthen predictive models and unravel causal mechanisms more precisely, but these data are currently lacking. We therefore joined the scientific consensus calling for more research across spatial scales [5, 53, 54] and examined leprosy disease dynamics at a broader scale where data on *M. leprae* and leprosy in Brazil were available, while acknowledging that sufficient long-term series remain scarce.

### Environmental and socioeconomic drivers of leprosy risk

Our Leprosy Risk in Humans Model highlights the critical role of environmental and socioeconomic factors in disease transmission. Variables such as higher rainfall seasonality, specific vegetation types, lower human population density, and altered habitats were found to contribute to the emergence of new leprosy cases. Rainfall seasonality influences human and armadillo behaviour, potentially affecting their interactions [55]. Habitat alterations, including deforestation and land conversion, may also increase human exposure to infected wildlife reservoirs by bringing communities into closer contact with these animals.

Lower human population density was also identified as a factor that may contribute to increased leprosy risk. In more sparsely populated areas, people are often isolated from large urban centrers where healthcare infrastructure is more accessible and robust. This isolation results in more prolonged human-to-human contact, reduced access to healthcare services and greater reliance on wildlife for food and livelihoods, intensifying human contact with potential zoonotic reservoirs. These findings underscore the need to integrate environmental, ecological, cultural, and socioeconomic factors into disease modelling efforts to more accurately predict and mitigate leprosy transmission risks.

### Armadillo hunting and leprosy transmission

Armadillo hunting and consumption are widespread across Brazil and other parts of the tropical Americas [56, 57]. While hunting in Brazil occurs for multiple reasons—including hunger, recreation, and economic or cultural traditions—armadillo hunting is primarily associated with food insecurity and recreational hunting [18, 19, 21]. Our study focuses primarily on illegal sport hunting, excluding subsistence hunting, which may lead to an underestimation of areas where hunting occurs. However, previous research in Brazil indicates that the species targeted by both subsistence and sport hunters are largely similar, suggesting that our findings may still reflect broader hunting patterns [58]. Nevertheless, subsistence hunting increases human-wildlife contact and can potentially heighten the risk of leprosy and other zoonoses [21] and should be considered in further studies accordingly.

Global data on wild meat hunting suggests that in tropical regions across South America, Sub-Saharan Africa, and South and Southeast Asia, approximately 150 million households depend on wild meat as a primary source of protein [59]. Regions in northern Brazil within the Amazon, where malnutrition rates are high and healthcare access is limited, exhibited a particularly higher percentage of human leprosy attributable to armadillo hunting [60]. In these areas, both reliance on wild meat and links with rural culture can pose significant public health challenges, especially in underserved areas far from primary healthcare centrers. These areas should be considered critical hotspots for further research and targeted interventions aimed at reducing zoonotic spillover and limiting disease transmission.

Comparisons with data from the United States—a non-endemic country—and Brazil suggest that armadillo hunting significantly contributes to human leprosy cases [18]. In our study, we found that at least a quarter of general cases in Brazil can be attributed to armadillo hunting. Municipalities in the Amazon had particularly high rates of leprosy linked to armadillo contact, with 40% of human cases associated with hunting activities in some large areas of Rondônia, Mato Grosso, Pará and Maranhão states, which present high deforestation rates and high prevalence of *M. leprae* infections in armadillos. These findings reinforce the hypothesis that zoonotic transmission plays a major role in sustaining high leprosy prevalence in certain regions of Brazil.

Beyond armadillos, other mammal species have been identified as potential reservoirs of *M. leprae*. Recent studies found that possums and certain rodents may carry the bacterium, raising concerns about alternative transmission pathways [61, 62]. A study of road-killed armadillos found *M. leprae* in 42% of sampled individuals, marking the first detection of the pathogen in the greater naked-tailed armadillo (*Cabassous tatouay*) [29]. However, another study found zero prevalence of *M. leprae* in various armadillo species in the Pantanal and Cerrado [63]. More recently, researchers identified *M. leprae* in multiple mammal species in a hyperendemic region of Brazil, including commonly hunted species such as deer and capybara [64]. While our study emphasizes the hunting of specific armadillo species, these findings highlight the importance of considering a broader range of potential hunted hosts in understanding leprosy transmission dynamics.

The presence of *M. leprae* DNA in soil and water samples from endemic regions further complicates the understanding of transmission pathways, suggesting that other environmental reservoirs may contribute to human infections [65]. Although the role of environmental transmission has not been fully demonstrated, the frequent presence of humans in shared habitats with armadillos and other potential hosts during hunting activities could contribute to leprosy transmission. Additionally, due to the long incubation period of leprosy, tracing the exact moment and mechanism of infection remains difficult. Many individuals diagnosed with the disease are unable to identify their index case or ‘source of infection’, indicating the need to investigate environmental sources of *M. leprae* and better integrate animal-human–environment transmission into leprosy control strategies [66, 67].

### Public health implications and the need for integrated actions

Since 1991, Brazil has participated in the WHO agreement to reduce and control leprosy cases, leading to the implementation of public health policies aimed at improving early diagnosis, strengthening surveillance, and expanding multidrug therapy accessibility. These initiatives have helped reduce the number of new cases detected annually. However, despite these efforts, recent studies show that leprosy remains hyperendemic in the North and Midwest regions and highly endemic in the Northeast [68].

The limited understanding of zoonotic spillover mechanisms for *M. leprae* raises concerns about latent epidemic outbreaks of leprosy. Historically, environmental disturbances, including habitat degradation and overhunting, have been linked to pathogen spillover, affecting host population dynamics, and increasing human exposure to disease reservoirs [69]. Our results support this, showing that leprosy prevalence in both armadillos and humans is lower in regions with higher natural habitat coverage and that leprosy rates are significantly higher in areas with severe environmental degradation, such as the “arc of deforestation” in the Cerrado-Amazon frontier. In these deforested regions, large species usually targeted by hunters are extinct or at low numbers, making smaller mammals such as armadillos a suitable alternative target, amplifying zoonotic transmission risks [31]. Addressing these risks requires proactive interventions to reduce public health vulnerabilities in zoonotic emergence hotspots [70], including comprehensive and integrative public health strategies [32, 71]. This should also involve greater investments in surveillance and enforcement to combat the synergistic effects of illegal hunting and environmental degradation [70, 72].

The WHO's Global Leprosy Strategy (2021–2030) calls for the development of integrated, country-specific roadmaps to achieve a leprosy-free world, listing zoonotic reservoirs as one of the research topics of key importance [15]. Given our findings—showing substantial zoonotic transmission potential—a sole focus on human-to-human transmission may be insufficient to control the disease. Accordingly, the intersection of hunting activities and infectious disease dynamics in the Americas demands urgent attention. This concern is particularly pressing, as hunting—spanning from the icecaps to the pantropics—intensifies human-wildlife interactions, heightening the risk of zoonotic spillover and disease transmission. Addressing this issue requires a critical reassessment of mode and pathway transmission mechanisms and a re-evaluation of the ecological and public health implications of established hunting practices at both local and global scales [73, 74].

More effective interventions should address reducing armadillo hunting activities where possible and enhancing health communication strategies to increase awareness of zoonotic risks and promote adequate safety measures while hunting, handling and consuming armadillos. These actions align with the UN’s Sustainable Development Goals, particularly those aimed at improving health, reducing poverty, and fostering sustainable environmental practices. Additionally, genetic studies are needed to directly link *M. leprae* strains in wildlife reservoirs to human infections in Brazil. Given the ongoing impacts of climate change [75] and land-use transformation, preserving natural habitats will also be crucial in reducing human exposure to emerging zoonotic diseases and preventing future outbreaks [76].

## Conclusions

This study highlights the urgent need for targeted public health interventions and continued research into ecological and socioeconomic factors driving leprosy transmission in Brazil and the wider Americas. It also strengthens the field of disease ecology by deepening our understanding of the complex interactions between wildlife use, environmental change, and zoonotic disease emergence. Although leprosy remains treatable, the synergistic effects of environmental degradation, illegal, unsustainable, and unsafe hunting practices, and zoonotic spillover present a growing public health threat, necessitating sustained surveillance and policy measures to safeguard vulnerable human populations and prevent future outbreaks.

## Supplementary Information

Additional file 1.

Additional file 2.

Additional file 3.

## Data Availability

The datasets analysed during the current study available from the corresponding author on reasonable request.

## Abbreviations

AUC: Area under the “receiver operating characteristic (ROC)” curve
VIF: Variance inflation factor
AIC: Akaike information criterion
GLM: Generalised linear model
GLMM: Generalised linear mixed model
WHO: World Health Organization
NTD: Neglected tropical disease
