# Supplementary material for: Wildlife hunting and the increased risk of leprosy transmission in the tropical Americas: a pathogeographical study
Source: Infect Dis Poverty. 2025 May 12;14:38. doi: 10.1186/s40249-025-01301-z (PMC12067952; doi:10.1186/s40249-025-01301-z)
Supplement: Supplementary file 1 — Additional file 1. [file 40249_2025_1301_MOESM1_ESM.docx]

**Supplementary Information**

**Wildlife hunting and the increased risk of leprosy transmission in the tropical Americas: a pathogeographical study**

**Table of Contents**

**Table S1**. Predictor variables considered in the Armadillo Hunting Model, Armadillo Leprosy Model and Risk of Leprosy in Humans Model.

**Table S2**. Armadillo Hunting Model outputs.

**Table S3.** Evaluation of the Armadillo Hunting Model and the distribution models used to build it based on discrimination and classification capabilities.

**Table S4.** Armadillo Leprosy Model outputs.

**Fig. S1.** Maps showing the occurrence records of *Dasypus* spp. and *Euphractus sexcinctus* in Brazil.

**Fig. S2.** Distribution models and hunting models of *E. sexcinctus* y *Dasypus* spp.

**Table S1**. Predictor variables considered for modelling. Some variables were used only in specific models: Armadillo-Hunting Model*, Leprosy Armadillo Model** and Human Leprosy Risk Model***

| **Factor** | **Code** | **Variable** | **Source** |
| --- | --- | --- | --- |
| Climate | *Bio1* | Annual Mean Temperature | Chelsa (http://chelsa-climate.org) |
|  | *Bio5* | Max Temperature of Warmest Month |  |
|  | *Bio6* | Min Temperature of Coldest Month |  |
|  | *Bio7* | Temperature Annual Range (Bio5-Bio6) |  |
|  | *Bio12* | Annual Precipitation |  |
|  | *Bio15* | Precipitation Seasonality (Coefficient of Variation) |  |
| Infrastructures | *Roads_km2* | *Density of roads* | Extension of state and federal roads (km; dated from 2021 and sourced from Departamento Nacional de Infraestrutura de Transportes:https://www.gov.br/dnit/pt-br), divided by the municipality area. |
| Socioeconomic | *Log_hpd_2021**** | Human population density (log) | Brazilian Institute of Geography and Statistics (IBGE, 2021)  IBGE. (2021). Estimativas populacionais para os municípios e para as Unidades da Federação brasileiros 2021. Available at: https://www.ibge.gov.br/. |
|  | *Malnutrition**** | Malnutrition rate | Reports on the nutritional status of individuals monitored by period, life cycle stage, and index in the year 2021 (ages 0 to 5 years). Available at: https://sisaps.saude.gov.br/sisvan/relatoriopublico/index#) |
|  | *IDHM_2010**** | Índice de Desenvolvimento Humano Municipal (Municipality Human Development Index) | Brazilian Human Development Atlas (PNUD, 2013),  PNUD - Brazilian Atlas of Human Development. 2013. Ranking IDH Municípios. Available at: http://www.atlasbrasil.org.br/2013/en. |
|  | *Phone_cover*** | Phone cover in Brazil | Sourced from Agência Nacional de Telecomunicações https://www.gov.br/anatel/pt-br/dados/qualidade/qualidade-dos-servicos/cobertura-da-telefonia-movel ) |
|  | *Log_PIB_per_capita**** | GDP per capita | Brazilian Institute of Geography and Statistics (IBGE, 2021). Produto Interno Bruto dos Municípios - 2021. |
|  | *POV_2010**** | Poverty rate | Brazilian Human Development Atlas (PNUD, 2013),SOURCE  PNUD - Brazilian Atlas of Human Development. 2013. Ranking IDH Municípios. Available at: http://www.atlasbrasil.org.br/2013/en |
|  | *B_votes**** | Percentage of people voting for Bolsonaro in 2022 | Percentage of votes for defeated and former president Jair Bolsonaro in the 2nd round of presidential elections in 2022 (sourced from Tribunal Superior Eleitoral, available at<https://www.tse.jus.br/eleicoes/eleicoes-2022>), |
|  | *Gun_ind** | *Proportion of guns per civilian* | Obtained through direct request from the Brazilian Federal Police (Number of guns in the municipality divided by the total population of the municipality) |
| Hydrography | *HydricAvai*** | Hydric availability | https://metadados.snirh.gov.br/geonetwork/srv/por/catalog.search#/metadata/7ac42372-3605-44a4-bae4-4dee7af1a2f8 |
|  | *Rivers_km2*** | Density of navigable rivers | Length of navigable rivers (km; 2017) sourced from the River and Groundwater Basins of the World, BGR/UNESCO (<https://www.whymap.org/whymap/EN/Maps_Data/Rgwb/rgwb_node_en.html>); |
| Ecosystem Types | Class 30 | Mosaic Vegetation (grassland, shrubland, forest) (50-70%) / Cropland (20-50%) | GlobCover (GC) Land Cover version 2.3 database for 2009 [1] |
|  | *Class 40* | Closed to open (>15%) broadleaved evergreen and/or semi-deciduous forest (>5m) |  |
|  | *Class 50* | Closed (>40%) broadleaved deciduous forest (>5m) |  |
|  | *Class 60* | Open (15-40%) broadleaved deciduous forest (>5m) |  |
|  | *Class 70* | Closed (>40%) needleleaved evergreen forest (>5m) |  |
|  | *Class 90* | Open (15-40%) needleleaved deciduous or evergreen forest (>5m) |  |
|  | *Class 100* | Closed to open (>15%) mixed broadleaved and needleleaved forest (>5m) |  |
|  | *Class 110* | Mosaic Forest/Shrubland (50-70%) / Grassland (20-50%) |  |
|  | *Class 120* | Mosaic Grassland (50-70%) / Forest/Shrubland (20-50%) |  |
|  | *Class 130* | Closed to open (>15%) shrubland (<5m) |  |
|  | *Class 140* | Closed to open (>15%) grassland |  |
|  | *Class 150* | Sparse (>15%) vegetation (woody vegetation, shrubs, grassland) |  |
|  | *Class 160* | Closed (>40%) broadleaved semi-deciduous and/or evergreen forest regularly flooded - Saline water |  |
|  | *Class 170* | Closed (>40%) broadleaved semi-deciduous and/or evergreen forest regularly flooded - Saline water |  |
|  | *Class 180* | Closed to open (>15%) vegetation (grassland, shrubland, woody vegetation) on regularly flooded or waterlogged soil - Fresh, brackish or saline water |  |
|  | *Class 200* | Bare areas |  |
|  | *Class 220* | Permanent snow and ice |  |
| Habitat | *Forest loss* | Non intact forest | High-Resolution Global Maps of 21^st^-Century Forest Cover Change [2] |
|  | *Natural_habitat (2019)* | Native habitat coverage | Remaining native habitat cover in 2019 (%; MapBiomas 2019) |
|  | *Veg_height* | Vegetation height | GEDI-Landsat data [3]. |
|  | *Biomass_AG* | Biomass Above Ground. | https://daac.ornl.gov/GEDI/guides/GEDI_L4B_Gridded_Biomass.html |
| Spatial descriptors | *Yesp3* | Spatial trend | Linear combination of spatial variables derived from continental-scale trend surface analyses [4] |

**References:**

[1] Bontemps S. GLOBCOVER 2009 Products Description and Validation Report. 2011.

[2] Hansen MC. High-resolution global maps of 21st-century forest cover change. Science. 2013;342: 850–853

[3] Potapov, P.; Hansen, M.C.; Kommareddy, I.; Kommareddy, A.; Turubanova, S.; Pickens, A.; Adusei, B.; Tyukavina, A.; Ying, Q. 2020. Landsat Analysis Ready Data for Global Land Cover and Land Cover Change Mapping. Remote Sens. 12, 426. https://doi.org/10.3390/rs12030426

[4] Legendre P. Spatial autocorrelation: Trouble or New Paradigm? Ecology. 1993;74: 1659–1673

**Table S2**. Armadillo Hunting Model outputs.

| ***Dasypus* spp.** | | | | |
| --- | --- | --- | --- | --- |
| **Variable** | **Estimate** | **Std. Error** | **Wald** | **p-value** |
| Constant | -22.937 | 7.821 | 8.601 | 0.003 |
| Favourability of species distribution | 1.072 | 0.661 | 2.629 | 0.105 |
| Min Temperature of Coldest Month | 0.007 | 0.003 | 7.072 | 0.008 |
| Closed to open (>15%) broadleaved evergreen or semi-deciduous forest | 1.238 | 0.329 | 14.136 | <0.001 |
| Slope (average) | -0.264 | 0.094 | 7.806 | 0.005 |
| Density of roads | -1.406 | 0.555 | 6.423 | 0.011 |
| Proportion of guns per civilian | 0.950 | 0.217 | 19.076 | <0.001 |
| Human population density (log) | 1.175 | 0.112 | 109.910 | <0.001 |
| Spatial trend of hunting | 0.790 | 0.131 | 36.273 | <0.001 |

| ***Euphractus sexcinctus*** | | | | |
| --- | --- | --- | --- | --- |
| **Variable** | **Estimate** | **Std. Error** | **Wald** | **p-value** |
| Constant | -0.867 | 1.262 | 0.473 | 0.492 |
| Favourability of species distribution | 1.311 | 1.283 | 1.044 | 0.307 |
| Proportion of guns per civilian | 1.350 | .481 | 7.874 | 0.005 |
| Human population density (log) | 1.207 | .222 | 29.608 | <0.001 |
| Spatial trend of hunting | 1.496 | .225 | 44.093 | <0.001 |

**Table S3. Evaluation of the Armadillo Hunting Model and the distribution models used to build it based on discrimination and classification capabilities.**

Sens.: sensitivity; Spec.: specificity; TSS true skill statistic; CCR: correct classification rate; Underp.: underprediction rate; Overp.: overprediction rate and AUC: area under the receiver operator characteristic curve.

|  | **Sen.** | **Spec.** | **TSS** | **CCR** | **Underp.** | **Overp.** | **AUC** |
| --- | --- | --- | --- | --- | --- | --- | --- |
| ***Dasypus* spp. Distribution Model** | 0.588 | 0.653 | 0.241 | 0.637 | 0.171 | 0.643 | 0.674 |
| ***Euphractus sexcinctus* Distribution Model** | 0.665 | 0.601 | 0.266 | 0.613 | 0.108 | 0.735 | 0.685 |
| ***Euphractus sexcinctus* Hunting Model** | 0.721 | 0.721 | 0.442 | 0.721 | 0.003 | 0.980 | 0.798 |
| ***Dasypu*s spp. Hunting Model** | 0.677 | 0.762 | 0.439 | 0.758 | 0.020 | 0.882 | 0,783 |
| **Hunting Model** | 0.745 | 0.587 | 0.332 | 0.595 | 0.022 | 0.913 | 0.744 |

**Table S4.** Armadillo Leprosy Model outputs.

| **Variable** | **Estimate** | **Std. Error** | **t value** | **Pr(>\|t\|)** | **Wald** |
| --- | --- | --- | --- | --- | --- |
| Intercept | -116 | 4.59E-03 | -25273 | <0.001 | 6.39E+08 |
| Mosaic Forest/Shrubland (50-70%) / Grassland (20-50%) | -2.633 | 9.46E-05 | -27849 | <0.001 | 7.75E+08 |
| Hydric availability | -0.328 | 2.75E-06 | -119199 | <0.001 | 1.43E+10 |
| Native habitat coverage | -2.278 | 2.05E-05 | -111310 | <0.001 | 1.24E+10 |
| Density of navigable rivers | -18.2 | 1.33E-04 | -137027 | <0.001 | 1.88E+10 |
| Closed to open (>15%) broadleaved evergreen or semi-deciduous forest | -2.619 | 2.74E-05 | -95449 | <0.001 | 9.11E+09 |
| Slope (average) | -0.794 | 6.75E-06 | -117622 | <0.001 | 1.38E+10 |
| Max Temperature of Warmest Month (log) | 34.24 | 1.32E-03 | 26002 | <0.001 | 6.76E+08 |
| Temperature Annual Range (log) | 0.667 | 3.44E-05 | 19376 | <0.001 | 3.76E+08 |
| Closed (>40%) broadleaved deciduous forest | -5.495 | 6.25E-05 | -87976 | <0.001 | 7.74E+09 |
| Density of roads | -0.833 | 4.83E-05 | -17244 | <0.001 | 2.98E+08 |
| Mosaic Vegetation (50-70%) / Cropland (20-50%) | -1.433 | 2.10E-05 | -68208 | <0.001 | 4.65E+09 |

**Fig. S1.** Maps showing the occurrence records of *Euphractus sexcinctus* and *Dasypus* spp. in Brazil.
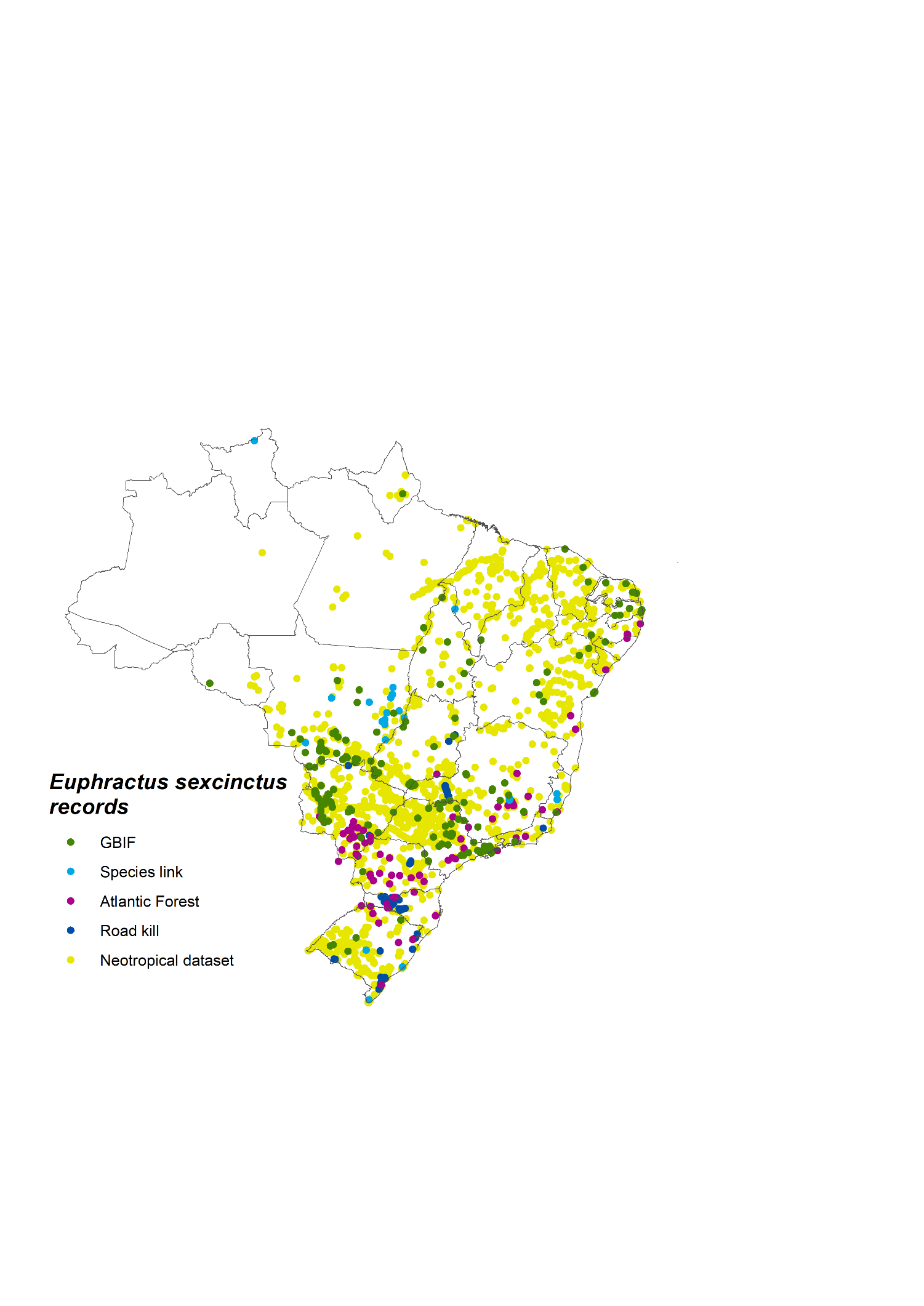


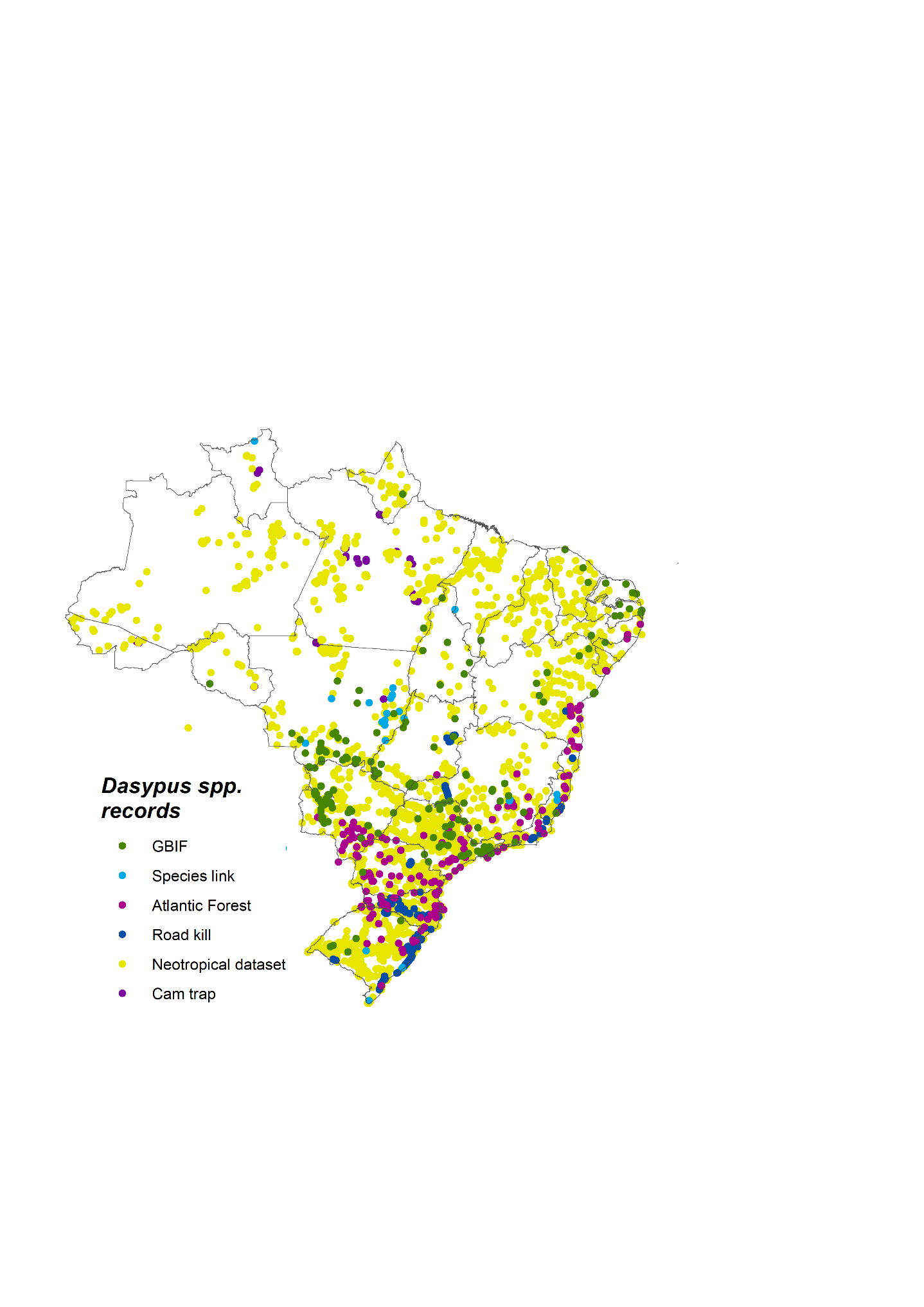


**Fig. S2.** Distribution models and hunting models of *E. sexcinctus* y *Dasypus* spp.

**
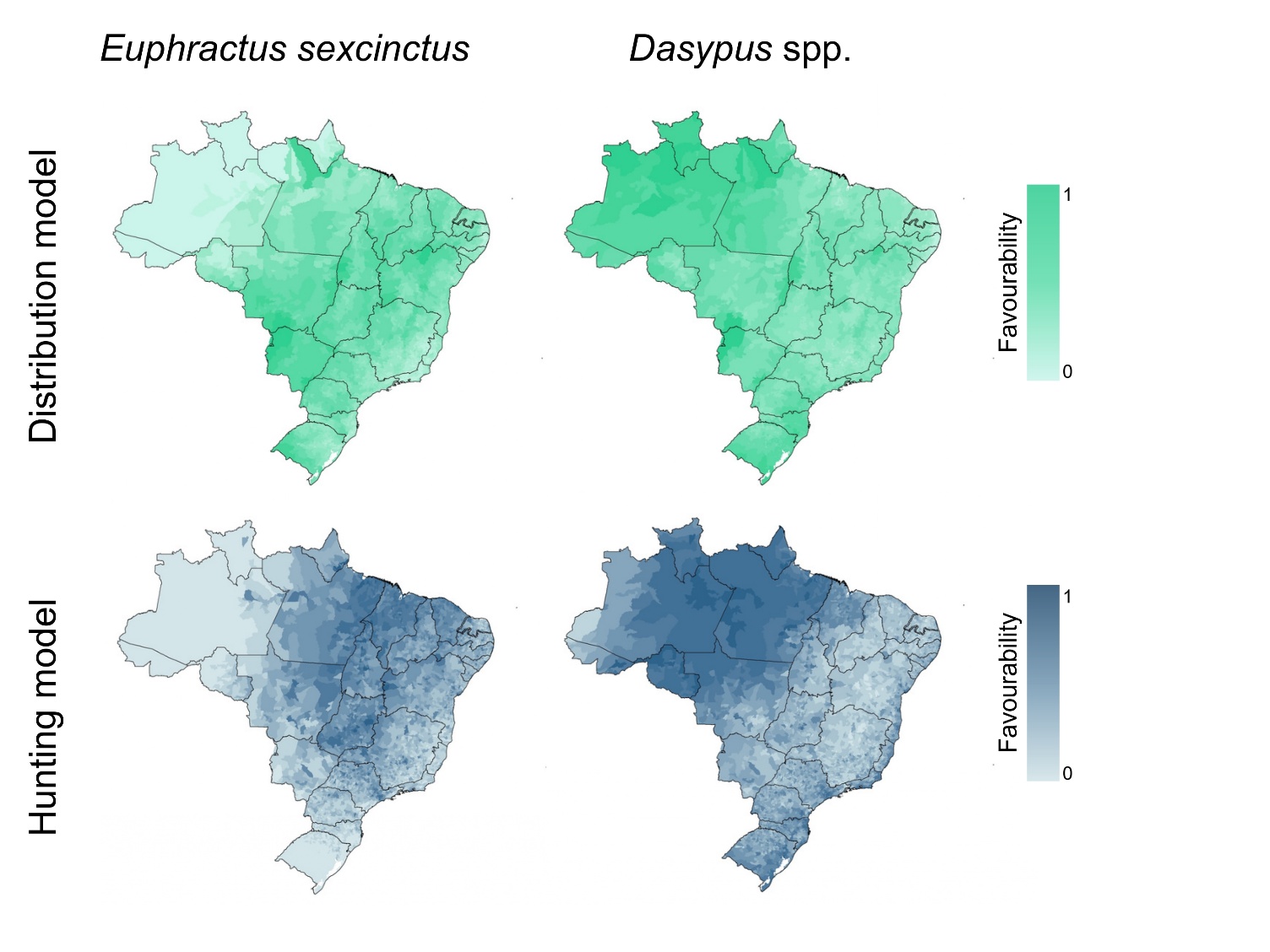
**
